# Optimal attentional modulation of a neural population

**DOI:** 10.3389/fncom.2014.00034

**Published:** 2014-03-26

**Authors:** Ali Borji, Laurent Itti

**Affiliations:** ^1^Department of Computer Science, University of Southern CaliforniaLos Angeles, CA, USA; ^2^Neuroscience Graduate Program, University of Southern CaliforniaLos Angeles, CA, USA; ^3^Department of Psychology, University of Southern CaliforniaLos Angeles, CA, USA

**Keywords:** top-down attention, neural modulation, neural coding, gain, tuning width, feature selectivity

## Abstract

Top-down attention has often been separately studied in the contexts of either optimal population coding or biasing of visual search. Yet, both are intimately linked, as they entail optimally modulating sensory variables in neural populations according to top-down goals. Designing experiments to probe top-down attentional modulation is difficult because non-linear population dynamics are hard to predict in the absence of a concise theoretical framework. Here, we describe a unified framework that encompasses both contexts. Our work sheds light onto the ongoing debate on whether attention modulates neural response gain, tuning width, and/or preferred feature. We evaluate the framework by conducting simulations for two tasks: (1) classification (discrimination) of two stimuli *s*_*a*_ and *s*_*b*_ and (2) searching for a target *T* among distractors *D*. Results demonstrate that all of gain, tuning, and preferred feature modulation happen to different extents, depending on stimulus conditions and task demands. The theoretical analysis shows that task difficulty (linked to difference Δ between *s*_*a*_ and *s*_*b*_, or *T*, and *D*) is a crucial factor in optimal modulation, with different effects in discrimination vs. search. Further, our framework allows us to quantify the relative utility of neural parameters. In easy tasks (when Δ is large compared to the density of the neural population), modulating gains and preferred features is sufficient to yield nearly optimal performance; however, in difficult tasks (smaller Δ), modulating tuning width becomes necessary to improve performance. This suggests that the conflicting reports from different experimental studies may be due to differences in tasks and in their difficulties. We further propose future electrophysiology experiments to observe different types of attentional modulation in a same neuron.

## 1. Introduction

Optimal neural coding, or efficient coding, suggests that sensory systems have evolved to optimize the representation of the world around us. Two seemingly different fields of study, neural coding and visual search, have addressed neural modulation. The former has mainly investigated the optimal tuning width for a population of neurons (often one value for all neurons) in stimulus reconstruction and discrimination tasks (e.g., Zhang and Sejnowski, 1999; Jazayeri and Movshon, 2006; Berens et al., 2011; Wang et al., 2012). For example the question of whether sharpening or broadening a neuron's tuning might improve performance has attracted significant interest (e.g., Pouget et al., 1999; Zhang and Sejnowski, 1999). Computational studies of top-down biasing of visual search, on the other hand, have primarily addressed optimal gain modulation (e.g., Navalpakkam and Itti, 2007; Scolari and Serences, 2009, 2010; Scolari et al., 2012). Optimal neural modulation, in general, is a complex optimization problem since several variables such as statistics of stimuli, task variability, limitations of neural systems (e.g., number of neurons and parameters, metabolic cost, noise), and coupled nonlinear dynamics are involved. Here, we present a reconciled and abstract account of optimal neural modulation by solving for the best set of gain, tuning width and preferred feature of individual neurons to maximize classification and visual search performance. We use terms *attention* and *optimal neural modulation* interchangeably since the term “attention,” as currently used in the literature, refers to a highly heterogeneous class of phenomena. Characteristics of these phenomena vary significantly depending on the specific context in which the nervous system is operating (e.g., different time scales, tasks, environments, etc.).

### 1.1. Overview of attentional modulation

Finding a friend amidst several hundred passengers at an airport can be a nightmare. Yet, our brain handles the explosion of information efficiently by filtering out irrelevant or distracting stimuli, and by drawing our gaze to salient and relevant visual stimuli, through a process known as visual attention (Treisman and Gelade, 1980; Tsotsos, 1992; Desimone and Duncan, 1995; James, 2011). Specifically, visual attention is believed to help in at least two ways: *goal-driven top-down attention* (Yarbus, 1967; Corbetta and Shulman, 2002; Borji and Itti, 2014) might help in focusing on relevant image regions that resemble our friend's appearance, thereby accelerating our search, and *stimulus-driven bottom-up attention* (Koch and Ullman, 1985) might alert us to salient image regions like moving cars, pedestrians or dollies in our way, thereby avoiding accidents (Itti and Koch, 2001). Together, top-down and bottom-up attention help us select a few relevant and salient image regions for further processing, including recognition, representation, awareness and action (Desimone and Duncan, 1995; Crick and Koch, 1998). Please see Itti and Koch (2001), Hayhoe and Ballard (2005), Macknik et al. (2008), Eckstein et al. (2009), Baluch and Itti (2011), Carrasco (2011), Eckstein (2011), Kowler (2011), Nakayama and Martini (2011), Schütz et al. (2011), Tatler et al. (2011), and Borji and Itti (2013) for recent reviews of attentional mechanisms at behavioral, computational, and neural levels.

There exists at least three types of attention – *spatial* (Posner et al., 1980; Moran and Desimone, 1985; Kastner et al., 1999; Womelsdorf et al., 2006; Talsma et al., 2007), *feature-based* (Treue and Trujillo, 1999; Saenz et al., 2003; Sohn et al., 2005; Maunsell and Treue, 2006; Serences and Boynton, 2007; Jehee et al., 2011) and *object-based attention* (Duncan, 1984, 1996; Roelfsema et al., 1998; Kanwisher and Wojciulik, 2000; Reynolds et al., 2003; Chen, 2012; Cohen and Tong, 2013), depending on whether the basic unit of attentional deployment is a spatial location/region (e.g., the attentional “spotlight” Treisman and Gelade, 1980; Crick, 1984; Brefczynski and DeYoe, 1999), visual feature (e.g., color, orientation), or an object.

Attention offers several behavioral advantages. It is known to:

- Improve processing of stimuli at the attended location (Posner et al., 1980),
- Improve detection of faint stimuli and to lower contrast thresholds (Carrasco et al., 2000; Baldassi and Verghese, 2005),
- Improve feature discrimination (Lee et al., 1999),
- Increase spatial resolution (He et al., 1996; Yeshurun and Carrasco, 1998),
- Reject unwanted stimulus noise (Lu and Dosher, 1998; Ling et al., 2009),
- Increase the rate of visual processing (Carrasco and McElree, 2001),
- Affect appearance (Liu et al., 2006).

In effect, attention filters out irrelevant stimuli from the visual input and enables neural resources to be focused on the relevant locations, features and objects (Zhang et al., 2011).

Attentional modulation is widespread in the brain and has been observed in multiple areas along the cortical hierarchy including:

- V1 (Motter, 1993; Watanabe et al., 1998; Martinez et al., 1999; Huk and Heeger, 2000; Saenz et al., 2002; Verghese et al., 2012),
- V2 (Motter, 1993; Luck et al., 1997),
- V4 (Haenny and Schiller, 1988; Spitzer et al., 1988; Motter, 1993; Connor et al., 1997; Luck et al., 1997; McAdams and Maunsell, 1999; Williford and Maunsell, 2006; David et al., 2008; Ipata et al., 2012),
- MT (Treue and Maunsell, 1996; O'Craven et al., 1997; Treue and Trujillo, 1999; Saenz et al., 2002; Sohn et al., 2005),
- Lateral Intra-Parietal cortex (LIP) (Bushnell et al., 1981; Colby et al., 1996; Gottlieb et al., 1998; Bisley and Goldberg, 2003),
- Frontal Eye Fields (FEF) (Bichot and Schall, 2002; Moore and Fallah, 2004; Bichot et al., 2005),
- Subcortical structures like Lateral Geniculate Nucleus (LGN) (O'Connor et al., 2002) and Superior Colliculus (SC) (Munoz et al., 1991; Fecteau and Munoz, 2006).

Attentional effects are task-dependent. In separate studies, attention to color/shape has been shown to enhance BOLD activity in V4, while attention in a speed discrimination task increases activity in MT, and attention in a contrast discrimination task increases activity in V1 (Corbetta et al., 1990; Beauchamp et al., 1997; O'Craven et al., 1997; Huk and Heeger, 2000; Verghese et al., 2012). In fact, simply instructing observers to pay attention to different aspects of a same stimulus on different blocks of trials triggers different observable attentional modulation effects, in distinct anatomical and functional cortical areas. For example, Watanabe et al. (1998) showed, using one stimulus with superimposed translating and expanding fields of dots, differential attentional modulation of BOLD activation, depending on whether the task was to attend to the translating or the expanding feature of the stimulus.

Although different neural mechanisms for attention have been reported, the physiology literature presently appears to be divided. Attention to a neuron's preferred location or feature could:

- Cause a leftward shift in the neuron's contrast response function thus increasing the effective contrast of the stimulus (Reynolds et al., 2000; Martinez-Trujillo and Treue, 2002),
- Increase the response gain of the neuron a.k.a multiplicative scaling (McAdams and Maunsell, 1999; Treue and Trujillo, 1999; Womelsdorf et al., 2008; Boynton, 2009; Reynolds and Stoner, 2009; Saproo and Serences, 2010; Scolari and Serences, 2010; Scolari et al., 2012),
- Decrease the neuron's tuning width a.k.a bandwidth scaling (Moran and Desimone, 1985; Haenny and Schiller, 1988; Spitzer et al., 1988),
- Increase neuron's baseline or spontaneous activity a.k.a additive scaling (Luck et al., 1997; Chelazzi et al., 1998; Chawla et al., 1999; Kastner et al., 1999),
- Shift neurons tuned to nearby locations toward the attended location (Connor et al., 1996; Womelsdorf et al., 2006; David et al., 2008; Ipata et al., 2012),
- Modulate neuronal interactions through neuronal synchronization (Fries et al., 2001; Womelsdorf and Fries, 2007; Womelsdorf et al., 2007).

**Figure 1:**
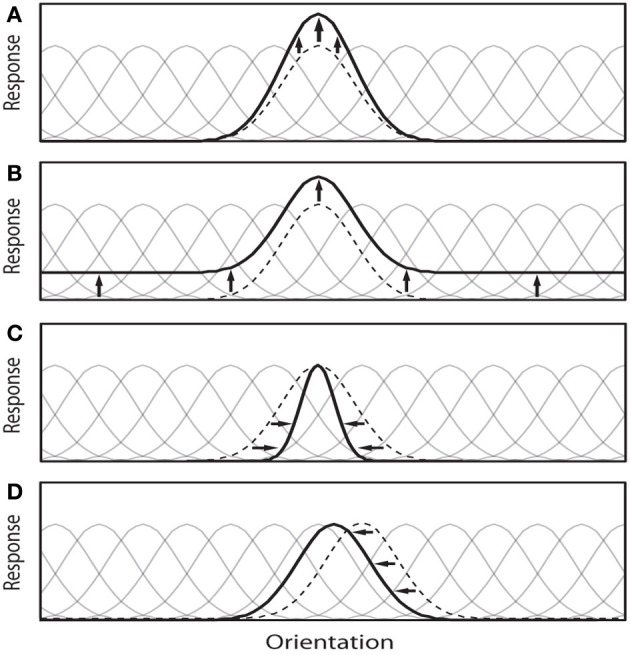
**This illustration depicts four possible attention-induced modulations of a neural population to a given visual task (here classification and visual search)**. Attention theoretically can: **(A)** Increase the gain of some important neurons a.k.a multiplicative scaling. This modulation selectivity increases the gain of the neurons that are more useful to find the target in visual search (or two classes in the classification and discrimination tasks). **(B)** Enhance response amplitudes in a feature-nonspecific manner a.k.a additive scaling. **(C)** Increase the selectivity of a neuron by modulating its tuning width (here sharpening) a.k.a bandwidth scaling, and **(D)** Shift tuning curves of neurons around to concentrate on important regions of the feature space (or shifting physical spatial receptive field of a neuron). Faint tuning curves correspond to the neural population before modulation, dotted black curve is the neuron under investigation, and the solid black curve is the modulated tuning curve. Here, we discard case **(B)** to make our simulations easier and tractable. Further, it has been argued that this case does not affect information decoding much.

Note that the underlying mechanisms responsible for these observed effects at the single-unit level may be more complex, for example involving biasing or winner-take-all (WTA) competitions among neurons in a local population (Desimone and Duncan, 1995; Lee et al., 1999), or through gain modulation of upstream neurons (McAdams and Maunsell, 1999). Figure 1 illustrates four possible types of attentional modulation of a neural population. Here, we discard the additive scaling since it has been argued that uniform translation of a tuning function does not affect the coding precision of that tuning function (Cover and Thomas, 1991) (but see Saproo and Serences, 2010), Paragraph 4 in the Discussion section and hence information content of a neural population. Further, this simplification makes our analysis easier and tractable.

### 1.2. Optimal attentional modulation

To gain better insight into above-mentioned discrepancies, we propose a unified account for optimal modulation of neural activity over two tasks: (1) *stimulus classification* (which of two stimuli was presented on the basis of the neural response pattern) and (2) *visual search* (i.e., enhancing the representation of the target stimulus, thus making search easier). Target selection often comes up in the context of a real world task such as visual search where the observer may be looking for a particular target, or for an unknown target that is the odd-ball. Our proposed framework can extend to additional tasks, including match-to-sample (as a neuron's response to the matching stimulus is enhanced while response to any non-matching stimulus is suppressed), discrimination, and stimulus reconstruction.

Let *p*(**r**|*s*_*a*_) and *p*(**r**|*s*_*b*_) be probability distributions of population activity **r** to two stimuli *s*_*a*_ and *s*_*b*_. The goal of optimal population modulation is to find the best set of parameters for each of *n* sensory neurons (i.e., θ_*i*_ = [*g*_*i*_, σ_*i*_, μ_*i*_] including gain, tuning width, and feature selectivity) such that:

(1) $$\phi^{*}=\underset{\phi }{\arg\max}f(p(r(\phi )|s_{a}),p(r(\phi )|s_{b})),\;\phi =[\theta_{i\;=\;1\ldots n}]$$

where *f* denotes the task objective function. For *classification* and *discrimination* tasks, *f* can be the mutual information between neural activity and behavioral response, or classification accuracy (e.g., linear discrimination error). Here we choose to maximize the inverse of minimum discrimination error (MDE) as the optimality criterion for the classification task. It has been shown that MDE has several advantages over other criteria such as Fisher Information (Berens et al., 2011). For *visual search* tasks, we choose to maximize signal to noise ratio (SNR). The concept of SNR has been suggested by psychophysicists as measured by the amount of overlap between target (=“signal”) and distractor (=“noise”) response distributions. If the purpose is *reconstruction* (i.e., estimate the true value of the presented stimulus on the basis of the noisy neural response **r**: *ŝ* = arg max_*s*_ *p*(*s*|**r**) ∝ arg max_*s*_ *p*(**r**|*s*)*p*(*s*)), then *f* can be the inverse of the mean squared error (MSE) between estimated stimulus (by means of a decoding method such as maximum-likelihood or population vector) and the actual input stimulus.

Optimizing above objective functions is a complex and time consuming process. For the brain this would be an optimization across many (usually thousands of) neurons, involving many different parameters which seems to be very daunting. Note that this does not happen instantly, rather it is a slow process of an organism learning to perform a task. Further, the stimulus distribution is also not available at once and demands the organism to interact with the environment and observe sensory data over time. Indeed, previous work by Baluch and Itti (2010) has shown that human observers become increasingly more efficient at biasing their visual system toward search targets in a triple conjunction search task. This suggests that humans learn over time how to bias the setting of their neural parameters so as to maximize task performance. Navalpakkam and Itti (2007) proposed a three-phase mechanism for learning top-down attentional modulation. In the first phase, bottom-up and top-down cues (learned previously) are applied to render some visual items salient. In the second phase, distributions of target and distractor features are learned through past trials, preview of picture cues, verbal instructions, etc. and in the third phase, optimal top-down gains (as well as other parameters) are computed (see Figure 2 in Navalpakkam and Itti, 2007). These gains will be later recalled and applied during future search trials.

## 2. Theoretical perspective

We formalize, in the Bayesian sense, how attention may modulate neural activity to optimize task performance. In classification tasks, the goal is to distinguish between a stimulus from class *C* = 1 [defined by a distribution of features *P*(*s*|*C* = 1) in some dimension such as orientation] from a stimulus from class *C* = −1 [defined by a distribution of features *P*(*s*|*C* = −1)]. In visual search, class *C* = 1 is considered the target *T* that is to be found among distractors *D* (*C* = −1).

We assume that the incoming visual display is processed by a population of *n* neurons tuned to different features. We further assume that all neurons have idealized and homogeneous tuning functions. Let **r**(*s*) = [*r*_1_(*s*), *r*_2_(*s*), …, *r*_*n*_(*s*)] denote the population vector of responses to input stimulus *s*. Assuming independent neurons, the probability distribution of response to a single stimulus *s* is:

(2) $$L_{r}(s)=p(r|s)=\prod_{j\;=\;1}^{n}p(r_{j}|s)$$

### 2.1. Classification

In classification tasks, a Bayesian ideal observer needs to estimate *Ĉ* = arg max_*C*_ *P*(*C*|**r**) = arg max_*C*_ *P*(**r**|*C*)*P*(*C*)/*P*(**r**) where *Ĉ* represents the estimated class (out of *m* classes). This equation means that the classifier chooses the class that was most likely to have caused the observed response pattern **r** on the basis of the stimulus conditional response distributions. For a two-class problem, the optimal neural decision variable depends on distributions of neural response to classes *P*(**r**|*C* = 1) and *P*(**r**|*C* = −1), each defined as:

(3) $$p(r|C)=\int p(r|s)p(s|C)ds=\int L_{r}(s)p(s|C)ds$$

Thus, to maximize classification performance, the MDE objective function (the error of the ideal observer model) tries to minimize the overlap between neural response distributions to the two classes:

(4) $$MDE(C=1,C=-1)=\frac{1}{2}\int \text{min}(p(r|C=1),p(r|C=-1))dr$$

Discrimination is a special case of classification, with *p*(*s*|*C* = 1) = *d*(*s* − *s*_*a*_) and *p*(*s*|*C* = −1) = *d*(*s* − *s*_*b*_), where *d* denotes the Dirac delta function. In Berens et al. (2011), authors have used MDE to solve for the optimal tuning width of a neural population in reconstruction and discrimination tasks.

### 2.2. Visual search

Assuming that attention during visual search is guided to locations of high neural activity, search performance can be optimized by maximizing the strength of the signal (expected total neural response to the target *C* = 1) relative to the noise (expected total neural response to the distractors *C* = −1). Thus, using the above formulas, SNR can be written as:

(5) $$\begin{array}{l} SNR(C=1,C=-1)=\frac{\sum_{i}E(r_{i}|C=1)}{\sum_{i}E(r_{i}|C=-1)}\\ \;=\frac{\sum_{i}\int r_{i}p(r_{i}|C=1)dr_{i}}{\sum_{i}\int r_{i}p(r_{i}|C=-1)dr_{i}}\\ \;=\frac{\sum_{i}\int \int r_{i}p(r_{i}|s)p(s|C=1)dsdr_{i}}{\sum_{i}\int \int r_{i}p(r_{i}|s)p(s|C=-1)dsdr_{i}} \end{array}$$

A closed-form solution for optimal gain modulation using SNR has been previously proposed in Navalpakkam and Itti (2007). Please note that here we attempt to solve visual search in feature space, irrespective of spatial organization of items in the search array. The SNR formulation has been shown to be capable of explaining a large number of psychophysics findings in the visual search literature (Verghese, 2001; Navalpakkam and Itti, 2007; Scolari and Serences, 2009, 2010; Jehee et al., 2011; Scolari et al., 2012). In addition, it has been shown that feature-based attention occurs independently of spatial attention (David et al., 2008), and feature-based attention changes activity globally throughout the visual-field representation (McAdams and Maunsell, 1999; Treue and Trujillo, 1999; Saenz et al., 2002; Maunsell and Treue, 2006; Serences and Boynton, 2007). In other words, attentding to a spatial location all features in that location are enhanced (McAdams and Maunsell, 1999; Boynton, 2009; Ling et al., 2009; Reynolds and Stoner, 2009). Conversely, attention to a specific feature results in global biases to that feature across the entire visual field (Treue and Maunsell, 1996; Treue and Trujillo, 1999; Saenz et al., 2002; Serences and Boynton, 2007).

## 3. Simulation results

We run two numerical simulations to investigate the optimal coding quality of a population of neurons under a range of stimulus conditions. The goal of this analysis is to reveal patterns or profiles of modulations depending on tasks and stimuli. Understanding how different patterns arise in different conditions can help design future experiments to pinpoint the neural basis of attentional modulation. In the first simulation, for simplicity and tractability, we choose a neural population of size 6 and we exhaustively search the parameter space for optimal solutions. We then run a second, larger simulation with 60 neurons on the most interesting cases. To illustrate our simulations, we consider the feature dimension of stimulus orientation, although our results apply interchangeably to other features such as color, spatial location, or direction of motion.

### 3.1. Small-scale simulation

We assume a conventional model of neural response, where the *i*-th neuron (*i* ∈ [1 *n*], in a population of *n* = 6 equi-spaced uncorrelated neurons in [0 180]) has a bell-shaped tuning function:

(6) $$\begin{array}{l} f_{i}(s)=g_{i}\times \left(\lambda_{1}+\lambda_{2}{\left(\frac{1}{2}+\frac{1}{2}cos(s-\mu_{i})\right)}^{20\sigma_{i}}\right);\\ p(r|s)=\frac{1}{\sqrt{2\pi \upsilon_{i}^{2}}}e^{-\frac{{\left(r\;-\;f_{i}(s)\right)}^{2}}{2\upsilon_{i}^{2}}} \end{array}$$

where *s* is the scalar stimulus feature (here orientation) and μ_*i*_ is the preferred feature of neuron *i*. The parameter *g*_*i*_ is the multiplicative gain. The parameter σ_*i*_ controls the width of the tuning curve. Large σ corresponds to steep tuning curves with small width. The parameters λ_1_ and λ_2_ set the baseline rate to 5 Hz and the maximal rate (amplitude) to 50 Hz. The firing activity of each neuron is assumed to follow a Gaussian distribution with Poisson-like noise, where variance is identical to mean spike count [i.e., υ^2^_*i*_ = *r*_*i*_(*s*) = 10*f*_*i*_(*s*)]. We estimate MDE and SNR (Equations 4, 5) using Monte Carlo techniques, by iteratively sampling from *p*(*s*|*C*), and, for each *s*, many times from *p*(*r*|*s*) to finally estimate *p*(*r*|*C*) (similar approach as in Berens et al., 2011).

We consider two types of constraint regimens on neural parameters. The *first regimen* constrains each free parameter to change only within a restricted window, to adhere to biophysical constraints. Note that, otherwise, in visual search, a trivial solution to optimize SNR would be for every neuron to shift its preference to the target feature, change its tuning to infinitely narrow, and enhance its gain infinitely. However, such unbounded changes would likely consume enormous energy (every spike is costly), would prevent neurons from adapting to dynamically changing environments, and are implausible given the electrophysiological observations described in the Introduction. Thus, to prevent indiscriminate changes leading to this mathematical singularity, we constrain each free parameter to change only within a restricted window. We set bounds for *g*_*i*_ to [0.5 2], for σ_*i*_ to [0.5 3], and for μ_*i*_ to [−0.2 0.2] (in radian, ~ 11.46°). A default value of 1 for *g*_*i*_ and σ_*i*_, and 0 for μ_*i*_ means no change.

Constraint regimen one imposes constraints at the single cell level. Another possibility is to consider constraints at the population level as suggested by Navalpakkam and Itti (2007) where the sum of each parameter over the neural population is constrained (Our *second regimen*, ∑*g*_*i*_ = 2, ∑σ_*i*_ = 3, and ∑μ_*i*_ = 2). This type of constraint needs more complex mechanisms to impose than constraint type one, for example by means of another neural network or a low-level molecular process. Similar to regimen one, regimen two leads to efficient spending of resources and energy but has more selective pressure as several solutions in regimen one may have equal objective function but in regime two optimization favors most informative neurons. Eventually, our treatment here is theoretical and further biological research is needed to discover which constraint is really implemented in the brain.

We also set the minimum value of *g*_*i*_ and σ_*i*_ to be 0.1 to preserve baseline activity. We employ real-valued Genetic Algorithms to exhaustively search the parameter space, in each individual dimension (i.e., *g* alone), for *g* + σ, as well as all three 3 parameters, to maximize SNR and MDE^−1^. It is worth noting that the qualitative conclusions derived from our simulations do not depend on the exact values of bounds.

**Figure 2:**
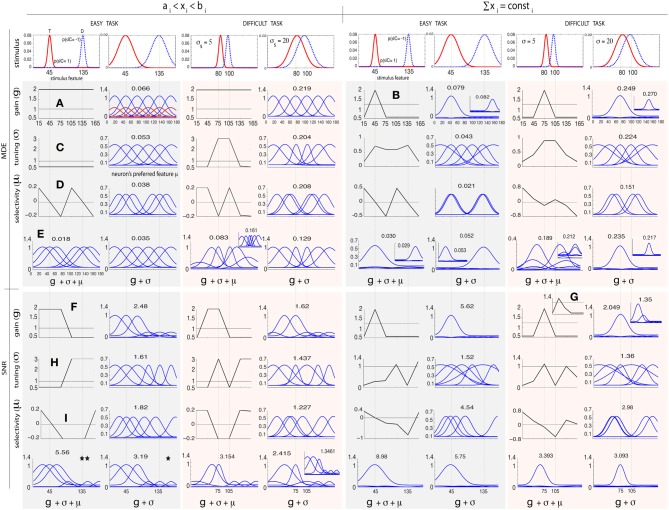
**Optimal attentional modulation for classification (MDE; top row) and visual search tasks (SNR; bottom row)**. The lower the MDE, the better (opposite is true for SNR). Left and right columns correspond to two parameter regimens for 2 classes of stimuli: (1) coarse classification (or easy search) *C* = 1 (target; solid line) at 45° and *C* = −1 (distractor; dashed line) at 135°, 2) fine classification (or hard search) *C* = 1 at 80°, and *C* = −1 at 100°, each at two uncertainty levels (σ_*s*_ = 5° and σ_*s*_ = 20°). **(A)** MDE, *g*, regimen 1: gains should be maximized for all neurons in both tasks. The red tuning curves represent the default tunings (σ = 1). **(B)** MDE, *g*, regimen 2: All gain is allocated to one of the two classes. **(C)** MDE, σ, regimen 1: all neurons in easy task should be widened. In other cases, neurons at 2 classes should be sharpened while the rest should be widened. **(D)** MDE, μ: neurons should be moved to locations of classes in all cases. **(E)** MDE, *g* + σ and *g* + σ + μ has the superposition of individual effects. **(F)** SNR, *g*, regimen 1: gains of neurons nearby target should be enhanced. In regimen 2, gain at the target should be amplified in easy task. **(G)** In difficult search (σ_*s*_ = 20°), the gain of the exaggerated neuron should be amplified more than the neuron at the target. **(H)** SNR, σ, easy task: neurons nearby target should be widened while neurons near distractor should be sharpened (see text). In difficult search task, neurons near target should be sharpened while neurons near distractor should be widened. **(I)** SNR, μ: neurons should be moved toward the target and away from the distractor.

**Figure 3:**
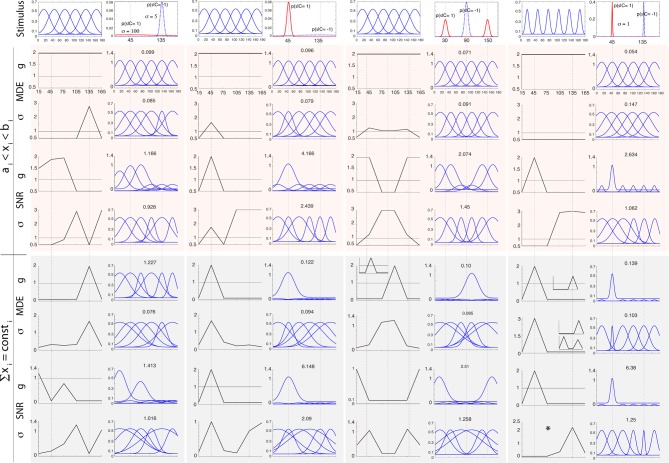
**Optimal neural modulation of *g*, σ and μ for additional cases mentioned in the text (small-scale simulation)**. Columns from left to right: unknown target (here modeled as a very wide distribution with σ_*s*_ = 100 shown with the dotted blue curve) and known distractor at 135° with σ_*s*_ = 5 (solid red curve), known target at 45° and unknown distractor, visual search for two targets at 30° and 150° and a single distractor at 90° with σ_*s*_ = 5, easy search for a target at 45° with a narrow distribution (σ_*s*_ = 1) and a distractor at 135°. In each column/setting, the left side shows the original neural population with 6 neurons and below that are the optimal parameters. The right side in each column shows the neural population after modulation. The top rows shows results for regimen 1 while the bottom one corresponds to regimen 2, for both classification (MDE) and visual search tasks (SNR). The panel with ^*^ shows the optimal σ in an easy visual search task.

Figure 2 shows simulation results obtained by modulating *g*_*i*_, σ_*i*_, and μ_*i*_ in the above manner for two arrangements of stimulus classes: (1) an easy task where two classes are far apart (*C* = 1 at 45° and *C* = −1 at 135°), and (2) a difficult task where two classes are close to each other and thus more similar (*C* = 1 at 80° and *C* = −1 at 100°). We investigate two levels of uncertainty (low σ_*s*_ = 5° and high σ_*s*_ = 20°) on stimulus distributions. For some cases in which solutions are not unique, we also show other good answers in insets. To further study the influence of stimulus distributions and initial parameterization, in Figure 3 we illustrate solutions to some additional cases: (1) when only knowledge about one class is known, (2) three classes of stimuli (two targets and one distractor; See Supplementary materials for heterogeneous search, i.e., one target among two distractors), and (3) narrow default tuning curves (σ_*i*_ = 5). In each test case, we first describe results for classification, then search.

#### 3.1.1. Response gain

In *classification*, under constraint regimen one, all neurons attain the maximum allowed gain, in both easy and difficult tasks. In regimen 2, all gains are concentrated around one of two classes, since both classes are equally important. Interestingly, and possibly counter-intuitively, if we were to distribute the gains equally around both stimulus classes, or equally among all neurons, the MDE would rise (i.e., worse classification). In *visual search*, SNR optimization shows that neurons tuned near the target feature undergo gain enhancement, while neurons tuned near the distractor feature undergo gain suppression (aligned with Treue and Trujillo, 1999 and Navalpakkam and Itti, 2007). While in regimen 2, only neurons at the target feature show gain enhancement, in regimen 1 neurons around the target are also enhanced. Interestingly in regimen 2, when target and distractor are very close and overlap is high (Figure 2F, *T* = 80°, *D* = 100°, σ_*s*_ = 20), in accordance with Navalpakkam and Itti (2007) and Scolari and Serences (2009), we also observe higher gain for the exaggerated neuron (at 45°) than for the neuron best tuned to the target (at 75°). However, unlike Navalpakkam and Itti (2007), baseline activity is sustained in our simulation, which agrees with electrophysiology findings (Chelazzi et al., 1998; Chawla et al., 1999; Kastner et al., 1999; David et al., 2008). Supporting single-unit evidence comes from feature-based attention tasks (McAdams and Maunsell, 1999; Treue and Trujillo, 1999; Martinez-Trujillo and Treue, 2004; David et al., 2008; Jehee et al., 2011).

#### 3.1.2. Tuning width

Maximum *classification* accuracy, in the easy task and in regimen 1, is obtained when all neurons widen their tuning as much as possible. In other cases (difficult task, regimen 1, and both tasks in regimen 2), optimization leads to sharpening near both stimuli and widening elsewhere (see also Figure 3). In *visual search*, our results suggest that attention causes both narrowing and widening of tuning width, and the choice depends on the difficulty of the task. In regimen 1, in the easy task, neurons at and near the target feature are maximally widened while neurons near the distractor feature are maximally sharpened. In regimen 2, in the easy task, we observe widening of neurons both at target and distractor, which was unexpected. Since neurons tuned near the distractor feature already respond strongly to the distractor (due to our bounds), sharpening would indeed only boost the distractor and lower SNR; however, widening for these neurons represents a “better worst-case scenario,” as it will make them respond to both distractor and target, resulting in slightly higher SNR compared to sharpening. When we made the task even easier (Figure 3^*^), we then observed that neurons at distractor sharpened. Over the difficult task in both regimens, we observe a sharpening at the target and widening near the distractor, which is the opposite of the easy task in regimen 1. When *p*(*s*|*T*) and *p*(*s*|*D*) do not overlap much (i.e., low uncertainty), and/or tuning curves are narrow and far apart, neural tuning widens near the target and sharpens near the distractor. The opposite happens when *p*(*s*|*T*) and *p*(*s*|*D*) highly overlap or the population is very dense. Note that parameter setting is important in the optimal answers. While exact values might differ for different parameter settings, we believe that patterns will stay the same (e.g., dependency of results to task difficulty). For experimental works, when biophysical properties of a neural population are known, it is easy to run a simulation (with our shared code) and verify a hypothesis. Supporting evidence for sharpening at the target comes from single-unit studies of orientation (Spitzer et al., 1988) and spatial tuning (Moran and Desimone, 1985).

#### 3.1.3. Preferred feature

In *classification*, optimization moves neurons toward either of the two classes as much as possible, in both regimens over both tasks. The optimal answer in *visual search* is to move neurons toward the target and away from the distractor. Supporting evidence for tuning shifts comes from single-unit studies in feature-based (David et al., 2008; Ipata et al., 2012) and spatial attention (Connor et al., 1996; Womelsdorf et al., 2006).

#### 3.1.4. All parameters

Comparing results obtained for the joint optimization of all parameters and the separate optimization of *g*, σ, and μ, we empirically find that the superposition of optimal answers to each individual parameter is always a good answer (although we do not have a theoretical guarantee on the optimality or uniqueness of such answer). For example, optimizing gain and tuning width jointly in easy visual search, regimen 1 (See Figure 2^*^), leads to maximal gain amplification and widening of neurons around the target, while minimizing gains of neurons selective to the distractor. Note that tuning width modulation of neurons near the distractor is not important here since their gain has already been minimized. When optimizing all three parameters, in addition to the joint answer of gain and tuning width, neurons are also shifted toward the target and away from the distractor (See Figure 2^**^). Our results also show that modulation of multiple parameters always yields better performance than optimizing only one or two parameters. This suggests that biological top-down attention may also affect multiple parameters, although most previous reports have focused on one parameter at a time.

Optimal neural modulation in heterogeneous visual search (i.e., one target among two distractors and vice versa) and optimizing *g*, σ, and μ with 12 neurons shows the same patterns as in Figure 2. These results are shown in Supplementary materials.

**Figure 4:**
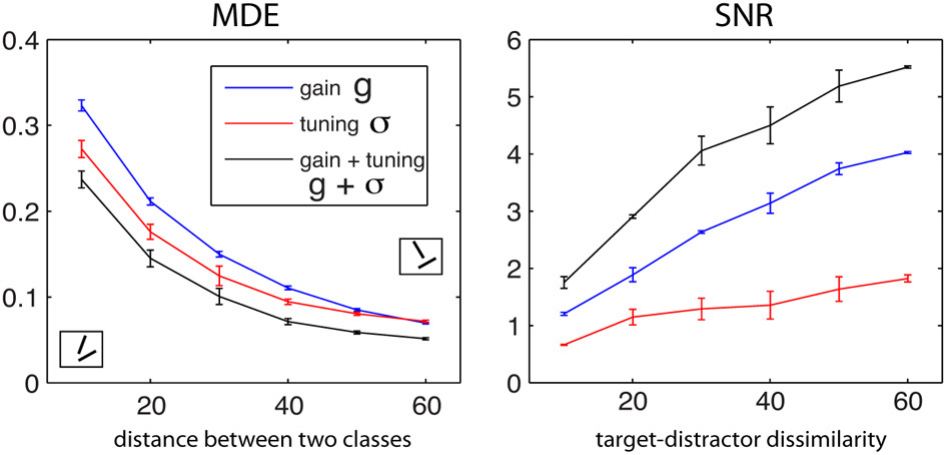
**Dependency of objective functions to dissimilarity between two classes for the small-scale simulation with 6 neurons for *g*, σ, and *g* + σ (averaged over *T* ∈ {30°, 40°, 50°, 60°} and *D* = *T* + {10°, 20°, 30°, 40°, 50°, 60°})**. Left: MDE for classification and Right: SNR for visual search. MDE decreases as two classes become more separate from each other while SNR raises which means that in both cases task becomes progressively easier.

Figure 4 shows the optimal MDE and SNR values (in regimen 1) as a function of target-distractor dissimilarity for *g*, σ, and *g* + σ (averaged over *T* ∈ {30°, 40°, 50°, 60°} and *D* = *T* + {10°, 20°, 30°, 40°, 50°, 60°}). Increasing the distance between the two classes leads to decrease in MDE and a ramp up in SNR. This qualitatively matches with human performance as a function of task difficulty (Duncan and Humphreys, 1989). Over both MDE and SNR, modulating both *g* and σ wins over single parameters. The tuning width is more effective than gain in classification, as seen by lower MDE values of σ than MDE values using *g*. The opposite occurs in visual search using SNR. One reason why SNR values for σ are small might be because neurons in this simulation are not allowed to sharpen beyond a certain limit.

#### 3.1.5. Note on noise correlation

In our simulations so far, we considered optimal modulation of an uncorrelated neural population for the sake of simplicity (i.e., uncorrelated noise). But, noise in the brain is correlated and this might influence the amount of information a neural population conveys (Averbeck et al., 2006) (See also Seriès et al., 2004 and Bejjanki et al., 2011). Here, we analyze the role of correlations (correlated noise) in optimal modulation of parameters for visual search (i.e., maximizing SNR) on our small scale neural population with 6 neurons.

Following Berens et al. (2011), we model the stimulus-conditional response distribution as a multivariate Gaussian:

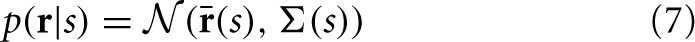



In above equation, **r**(*s*) = (*r*_1_(*s*), *r*_2_(*s*), …, *r*_6_(*s*)) and Σ(*s*) represent average spike counts and covariance matrix, respectively. This allows us to inject Poisson-like noise correlations into our simulation (See Berens et al., 2011 and their supplement for more details on adding correlated noise). Results are shown in supplementary materials for optimal answers of searching a target at 80° and distractor at 100° with σ_*s*_ = 5° (see Figure 2). We consider 10% noise correlation in our simulations. As it can be seen patterns of results are similar to those shown in Figure 2 for both constraint regimens and all three neural parameters. This could be because the effect of noise is vanished when averaging the neural activity, to targets and to distractors in SNR computation. For future research we encourage a more detailed look at noise correlations (e.g., non-uniform correlations) and how they may affect optimal solutions on larger neural populations.

### 3.2. Large-scale simulation

**Figure 5:**
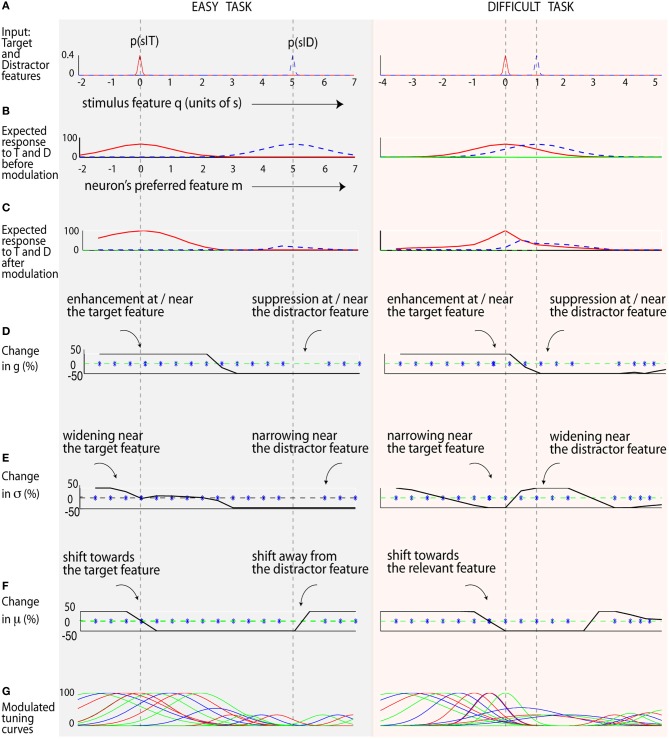
**Attentional modulation in easy and difficult visual search**. **(A)** The input stimuli. Rows **(B,C)** show the expected response of neurons (tuned to different features) before and after modulation. The solid red line is the expected response to the target, while the dotted blue line represents the expected response to the distractor. **(D)** The optimal shift in response gain is shown by the solid black line. Neurons tuned near the target increase their gain, while others tuned near the distractor undergo suppression. **(E)** The optimal shift in neuron's tuning width (σ) is shown here in the solid black line. In the difficult task, neurons tuned to the target feature decrease their tuning width, while nearby neurons widen their tuning width. **(F)** The optimal shift in preferred features μ is shown by the solid black line. A positive shift (Δμ_*i*_ > 0) indicates neurons shifting to the right, and vice versa. The blue star shows the neuron's preferred feature after the modulation. Neurons shift toward the target feature and away from the distractor feature (as seen by the lack of blue stars near the distractor). **(G)** The optimal tuning curves.

The previous analysis revealed different patterns of modulation depending on task and stimulus conditions. Importantly, it revealed that joint optimization of all parameters always yields better performance than optimizing only one parameter. This prompts us to study the relative utility or contribution of modulating each parameter as part of a joint optimization. To further investigate this, we focus on visual search in a larger-scale, more detailed simulation. We simulated a population of *n* = 60 equi-spaced, broad, overlapping Gaussian neurons with preferred stimulus feature μ_*i*_, tuning width σ_*i*_, amplitude λ_2_, gain factor *g*_*i*_, and baseline firing rate λ_1_:

(8) $$\begin{array}{l} f_{i}(s)=g_{i}\times \left(\lambda_{1}+\lambda_{2}e^{-{\left(s\;-\;\mu_{i}\right)}^{2}/2\sigma_{i}{}^{2}}\right)\text{​},\;i=1,\;\ldots ,\;n;\\ p(r|s)=\frac{e^{-f_{i}(s)}f_{i}{\left(s\right)}^{r}}{r!} \end{array}$$

with default tuning width of 10°, default gains at unity, spacing between preferred orientations of adjacent neurons 3° spanning 0–180° in orientation space (Figure 5). In addition, we consider the noise in neural response (to repeated presentations of a same stimulus) to have Poisson variability (used to numerically compute the expectations in the Equation 5). Here, we set λ_1_ = 0, for simplicity.

We jointly maximized SNR wrt. *g*_*i*_, μ_*i*_, and σ_*i*_ using a multi-start Nelder-Mead simplex algorithm (Nelder and Mead, 1965) (genetic algorithm was too slow in this larger-scale test). We used multiple initial conditions to avoid converging into local optima (20 different initial conditions, each with a random jitter in *g*_*i*_, μ_*i*_, and σ_*i*_ of up to 50% from default values), and considered the solution with maximum SNR. Here, attention can modulate *g*_*i*_ by up to ±50% of its default unity value, and σ_*i*_ and μ_*i*_ by up to ±50% of the default tuning width (corresponding to **regimen 1** and to avoid numerical instability).

Figure 5 shows how neural parameters may be optimally modulated in an easy search (with an orientation difference between target and distractors of 5σ_0_ = 50°), and a difficult search task (smaller orientation difference of σ_0_ = 10°). After modulation, the expected neural response to the target is much higher than the distractor (Figure 5C) compared to before modulation (Figure 5B). This effect is more clearly seen in the difficult task, where the initial population response to the target and distractor are similar (Figure 5B, 2nd column, hence a low SNR), but different after modulation (Figure 5C, 2nd column), leading to an improvement in SNR. Optimization results here are aligned with our smaller-scale simulation (Figure 2). Interestingly, since here target and distractor are well separated in the easy task, neurons around the target widen while those tuned near the distractor sharpen. In contrast, neurons sharpen near the target and widen near the distractor in the difficult task.

#### 3.2.1. Analysis of tuning curve overlap

**Figure 6:**
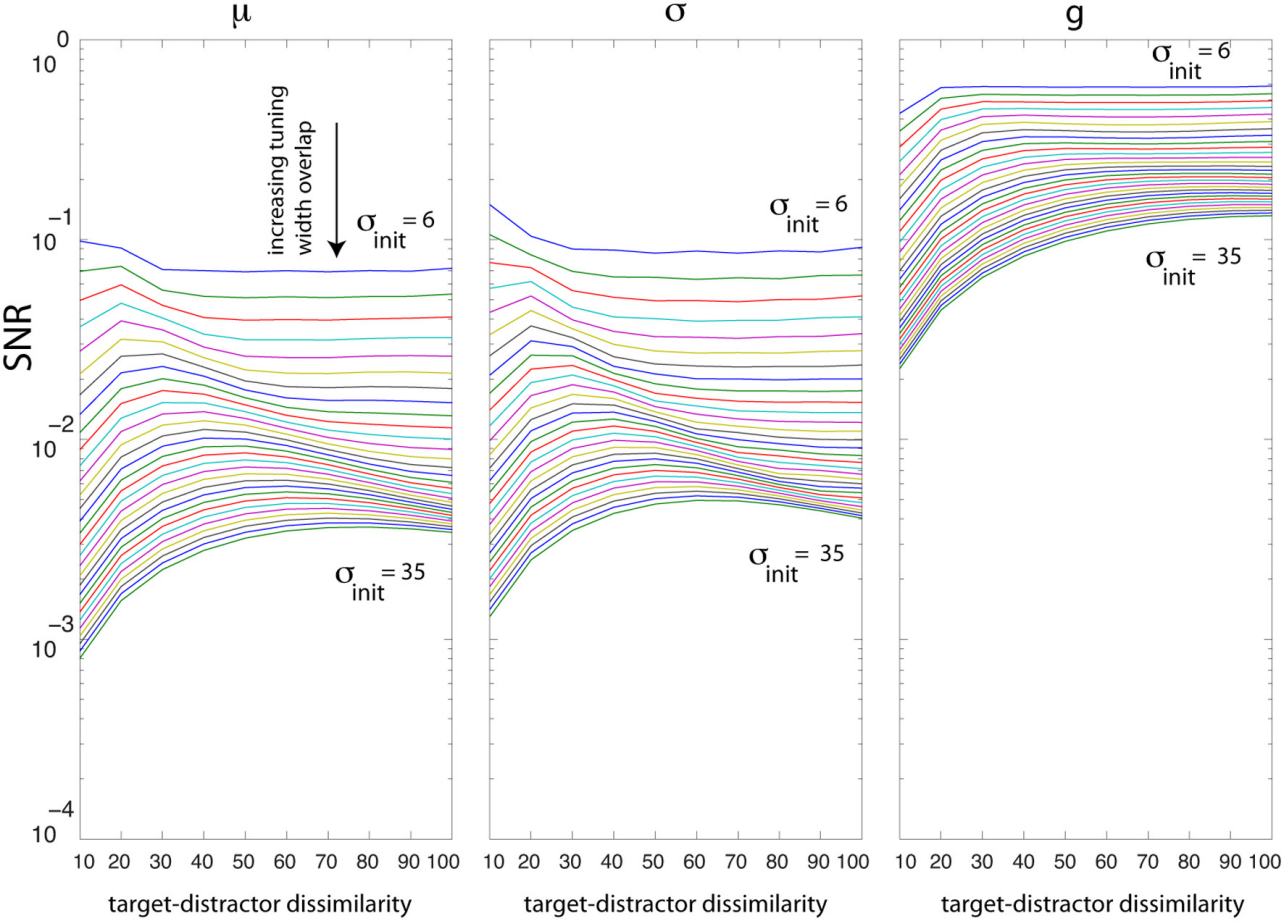
**Analysis of tuning curve overlap (σ from 6 to 35°; spacing between neurons is 3°)**. The *x* axis shows task difficulty due to target-distractor dissimilarity (measured by increasing orientation difference between the target and distractor: for *j* = 1:10, *T* = 60° − *j* × 5, *D* = 60° + *j* × 5). The *y* axis shows the best SNR achieved by optimizing each parameter. Curves from top to bottom indicate higher overlap between neurons. Increasing the neural overlap impairs the SNR due to optimal σ and μ more than SNR by *g*.

How much is SNR dependent on the degree of neural overlap? Over our population of 60 neurons, we change σ from 6° to 35° and task difficulty from 10° to 100° and then find the optimal solutions for *g*, σ, and μ. Figure 6 shows that increasing the overlap between neurons reduces SNR for all parameters regardless of task difficulty. This impairment is more profound in difficult tasks than in easy tasks. In easy tasks, irrespective of the degree of overlap, SNR values using gain are higher than SNR due to σ and μ. SNR using gain increases as the difference between target and distractor increases. Interestingly, there is an interaction between overlap and task difficulty when optimizing for σ and μ (non-monotonic curve shapes in Figure 6).

The analysis of SNR changes as a function of tuning overlap suggests explicit qualitative predictions that could be made when looking across cortical areas (given that orientation tuning inherently broadens as one ascends the visuocortical hierarchy). Moving along the hierarchy, neurons become broader (thus higher overlap among neurons) which eventually causes lower SNR. Also note that the peak of the curves in Figure 6 shifts to the right suggesting that maximum separability happens for more dissimilar stimuli.

#### 3.2.2. Behavioral utility of neural modulation

**Figure 7:**
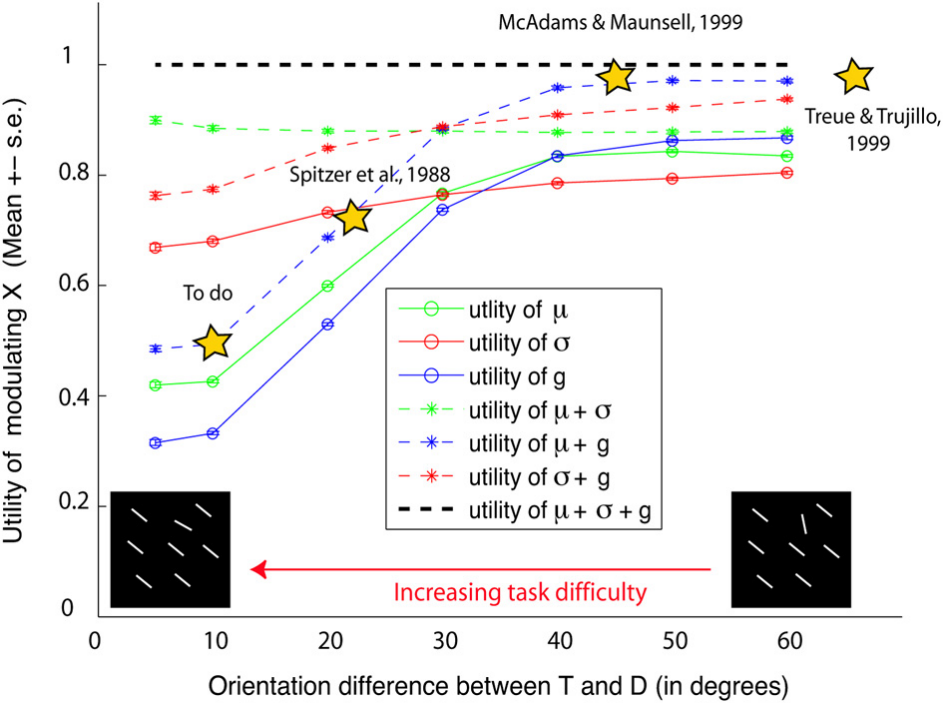
**Utility of attentional modulation**. The *x* axis shows task difficulty due to target distractor similarity. The *y* axis shows simulation predictions of utility of modulating preferred features (μ), tuning width (σ), response gains (g), or any combination of these parameters. For easy tasks, we predict that modulating preferred features and gains are useful and sufficient (yielding 0.97 × the best performance). But their combined utility decreases with decreasing orientation difference between the target and distractors (*u* = 0.49), rendering them less useful in difficult tasks. On the other hand, modulating tuning width is more useful and necessary in difficult tasks. A similar trend is observed in separately modulating gains or preferred feature vs. tuning width.

How useful is the modulation of each neural parameter? To answer this question, we computed a utility statistic *u*(*p*) for a parameter *p* ∈ {*g*, σ, μ} as the ratio of benefit to SNR obtained by modulating *p* alone vs. modulating everything. Higher utility values indicate that more performance is achieved by modulating *p* compared to other parameters, i.e., *p* is a high-yield parameter to modulate in the particular task and stimulus studied. As seen in Figure 7, *u*(*g*) and *u*(μ) both decrease with increasing task difficulty, but *u*(σ) does not. Thus, in easy tasks (where the target and distractor differ by Δ ≥ 40°) modulating *g* or μ is more useful, but becomes less useful in difficult tasks. On the other hand, while modulating σ is not very beneficial in easy tasks, it becomes necessary in difficult tasks (Δ ≤ 25°). Furthermore, in easy tasks, simulation predicts that the combined modulation of μ and *g* is sufficient to yield close to best behavioral performance, but their combined utility decreases with increasing task difficulty.

## 4. Discussion and conclusion

Results of two consistent simulations reveal that:

1. In classification, when two classes are well separated, all neurons should be widened and gains should be boosted,
2. In classification, when two classes are close in feature space, neurons selective to both should be sharpened and their gains should be increased,
3. In easy search, the optimal solution is to widen and boost gain at the target, and sharpen and reduce gain around the distractor (the opposite is seen for tuning width in difficult search),
4. Only in constraint regimen 2 and in difficult search, maximum gain is allocated to the exaggerated neuron as predicted by Navalpakkam and Itti (2007) and seen by Scolari and Serences (2009),
5. Feature selectivity of neurons should be biased toward target features (the two classes in classification) and away from distractors,
6. Optimizing multiple parameters is better than optimizing a single one and joint solutions seem to be combinations of constituent ones,
7. Increasing overlap among neurons worsens SNR, which is more harmful in difficult than in easy search,
8. Uniform noise correlation did not affect our conclusions but more detailed analysis of different noise conditions is encouraged,
9. Task difficulty is a key factor in determining the utility of a neural parameter.

Our theoretical investigation sheds new light on the ongoing controversy of attentional modulation, by indicating that the reported discrepancies in the literature may be due to differences in task difficulty (Figure 7). For instance, previous physiological studies that reported gain modulation (McAdams and Maunsell, 1999; Treue and Trujillo, 1999) used easy tasks: McAdams and Maunsell used an angular difference of 45° or 90° between target and distractor, while Treue and Martinez-Trujillo used either no distractor or one 180° from the target. Previous studies that found preferred feature modulation also used easy tasks: (Womelsdorf et al., 2008) used a spatial attention task where monkeys attended to a target location in the absence of distractors. In such easy tasks, as predicted by our theoretical analysis, modulation of gains and preferred features (which is most useful) is observed, while tuning width modulation (not useful) is not observed. One of the few previous studies (Spitzer et al., 1988) that reported tuning width modulation, observed it in more difficult discrimination tasks (smaller angular difference of 22.5°). Nevertheless, as tuning width modulation remains a controversial issue (e.g., Treue and Trujillo, 1999), our main goal here it to show how tuning width modulation is an optimal strategy when the task is difficult.

It is difficult to disentangle the effect of gain and tuning width modulation behaviorally (see Ling et al., 2009). We suggest neurophysiology experiments for this purpose by systematically controlling for task difficulty. An ideal task for testing tuning width modulation would be when the monkey attends to a target feature in the presence of flanking distractor (e.g., attend to a 45° oriented moving random dot pattern (RDT) among 50 and 40° oriented RDTs). In such a task, modulating preferred features or gains will not suffice as neurons responding to the target will also respond to similar distractors. Instead, sharpening the tuning curve will help the target-sensitive neurons by decreasing interference from distractors, hence better resolving the difference between target and distractor. In contrast, when the target and flanking distractor are very different (e.g., more than 45° apart), modulating tuning widths is not useful, and thus modulation of preferred features and gains should be observed.

Our model generalizes over previous gain-only models: guided search theory (Wolfe et al., 1989), feature-similarity gain principle (Treue and Trujillo, 1999; Martinez-Trujillo and Treue, 2004), and optimal gain theory (Navalpakkam and Itti, 2007). The guided search theory revises the feature integration theory (FIT) and suggests that top-down attention acts as a linear weighted combination of multiple features which in effect makes an object of interest more salient among distractors and decreases the search time. However, similar to FIT, this theory only attempts to explain the behavior of the organism. In the the feature similarity gain model, gain modulation is a function of similarity between the neuron's preferred feature and the target feature. This theory does not consider target-distractor similarity. The optimal gain theory, combines information from both the target and distracting clutter to maximize the relative salience of the target. Interestingly, this model predicts that it is sometimes optimal to enhance the non-target features (e.g., Figure 2G). Here, we considered three neural parameters and showed how distribution of target and distractors can be used to optimally tune all these parameters and make the target salient.

In addition to gain, our model offers testable predictions for tuning width modulation and shifts in selectivity (seen by David et al., 2008 and Ipata et al., 2012 in area V4). Our model differs from the well-established normalization model of attention (Reynolds and Stoner, 2009) in one main aspect: the normalization model commits to explain low-level attentional mechanisms, while our model offers a high-level theoretical account for optimal attention over a population of neurons, considering task difficulty, and stimulus statistics. Obviously, our model has limited prediction power. It may need to be further expanded to account for optimal spatial attention, when deployed jointly with feature-based attention in hybrid spatial/feature tasks. We encourage future neurophysiology studies, with our theoretical framework in hand, to further explore such tasks, which will give new insights for developing unified models of spatial and feature-based attention.

In summary, we investigated three attentional mechanisms, namely attentional modulation of neural response gain, tuning width and preferred feature. Reports from different laboratories differ on whether attention modulates tuning width or gain or preferred feature. We have proposed a simple computational model that reconciles the above differences by predicting that task-difficulty (due to target-distractor similarity) plays a critical role in determining attentional modulation. Our model predicts that gain and preferred feature modulation is useful in easy tasks, while tuning width modulation is useful in difficult tasks – a prediction that is in good qualitative agreement with reported data. This unified model illuminates the similarities and differences in reported data from various laboratories, and provides guidelines for future experiments.

### Conflict of interest statement

The authors declare that the research was conducted in the absence of any commercial or financial relationships that could be construed as a potential conflict of interest.
