# Supplementary material for: Optimal attentional modulation of a neural population
Source: Front Comput Neurosci. 2014 Mar 26;8:34. doi: 10.3389/fncom.2014.00034 (PMC3972484; doi:10.3389/fncom.2014.00034)
Supplement: Supplementary file 1 [file Presentation1.PDF]

5. Appendix A

Figure 8 shows the optimal attentional modulation for heterogeneous classification and visual search (i.e., a single target among two distractors) over a population of 6 neurons. Please see section 3.1.

6. Appendix B

Figure 9 shows simulation results over 12 neurons for classification and visual search. Please see section 3.1.

7. Appendix C

Figure 10 shows simulation results over 6 neurons with correlated noise in visual search. Please see the discussion section 3.1 for the explanation of results.

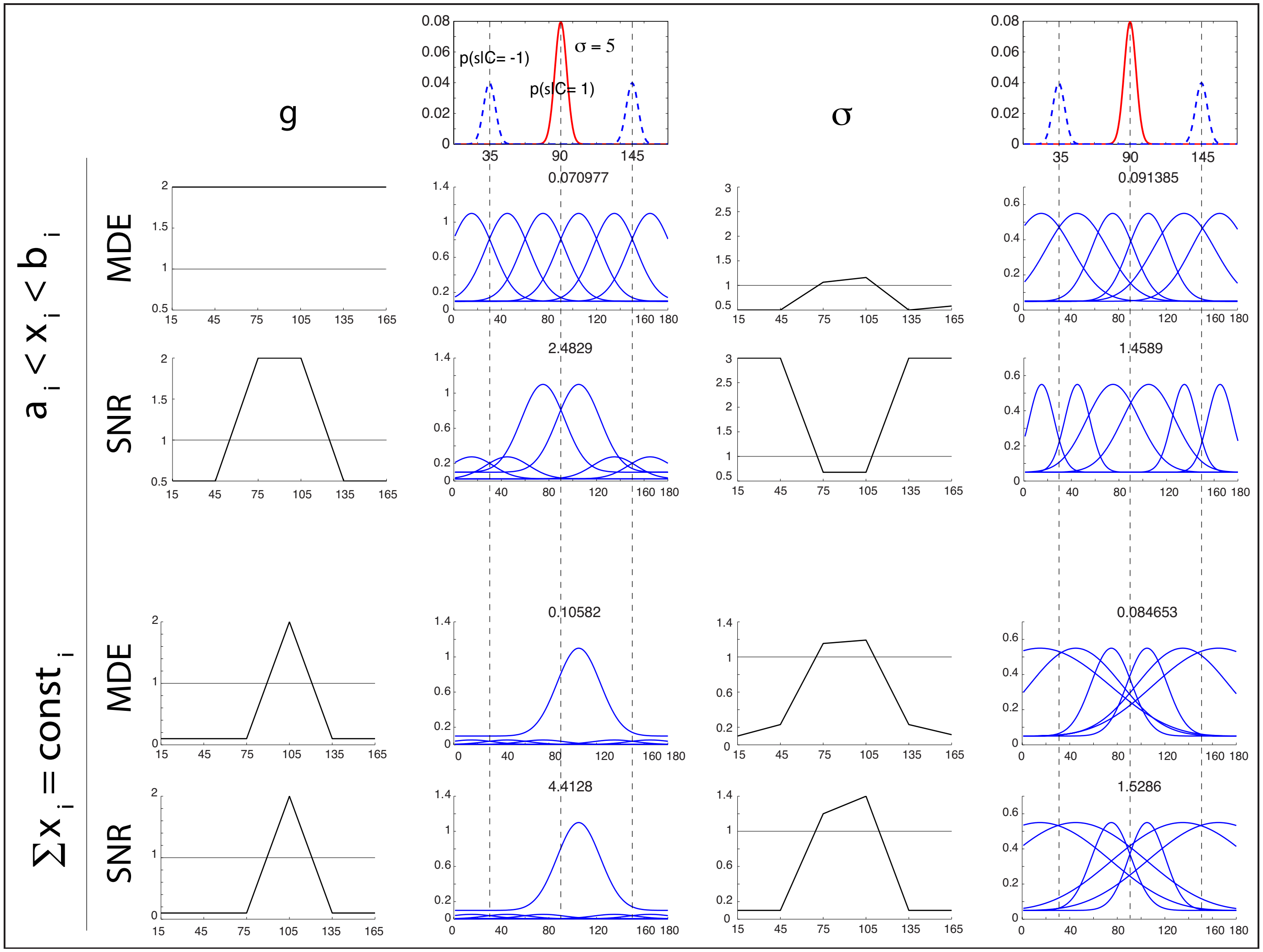

Figure 8: Optimal attentional modulation for heterogeneous classification and visual search (one target among two distractors) over a population of 6 neurons.

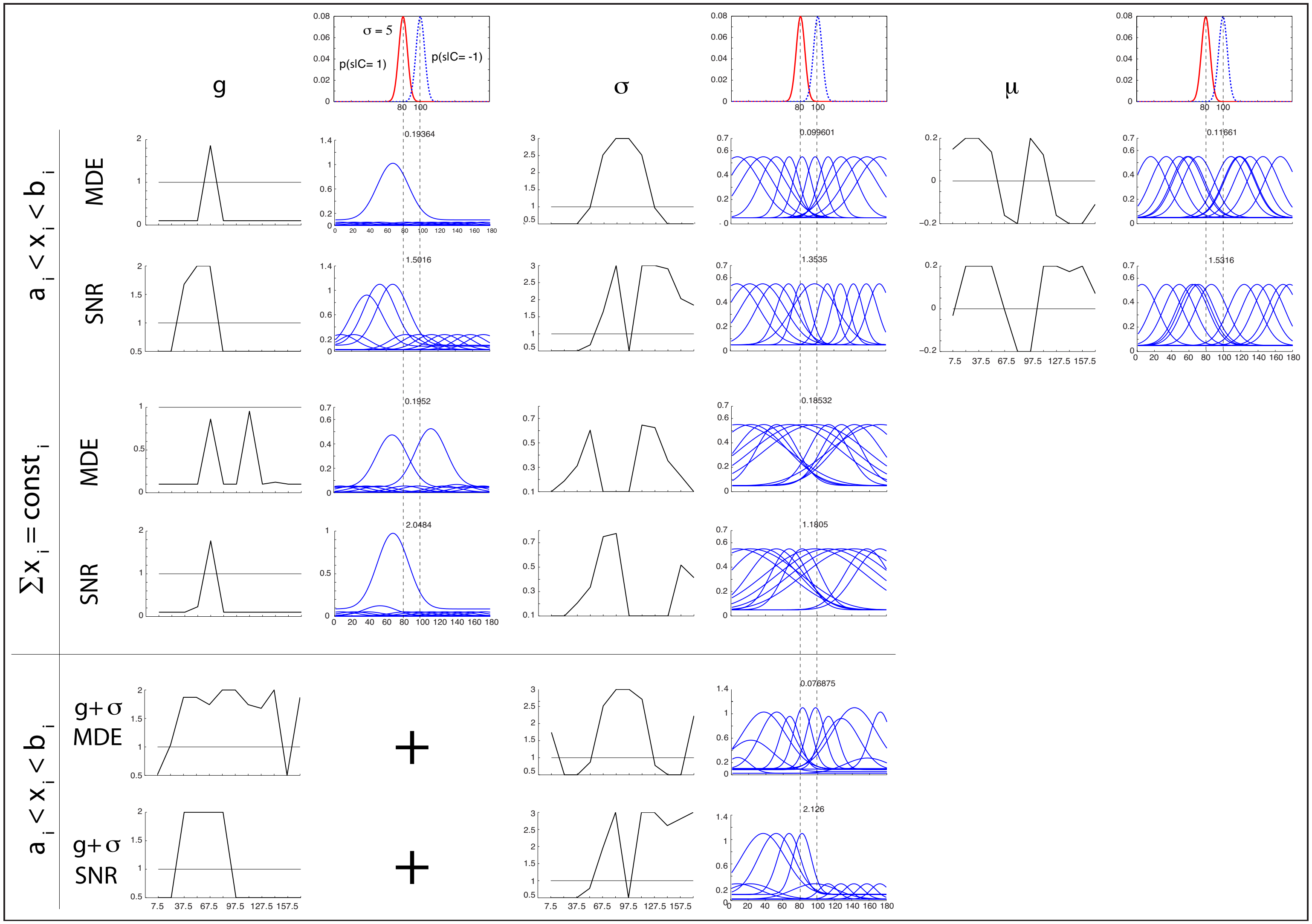

Figure 9: Simulation results over 12 neurons for classification and visual search.

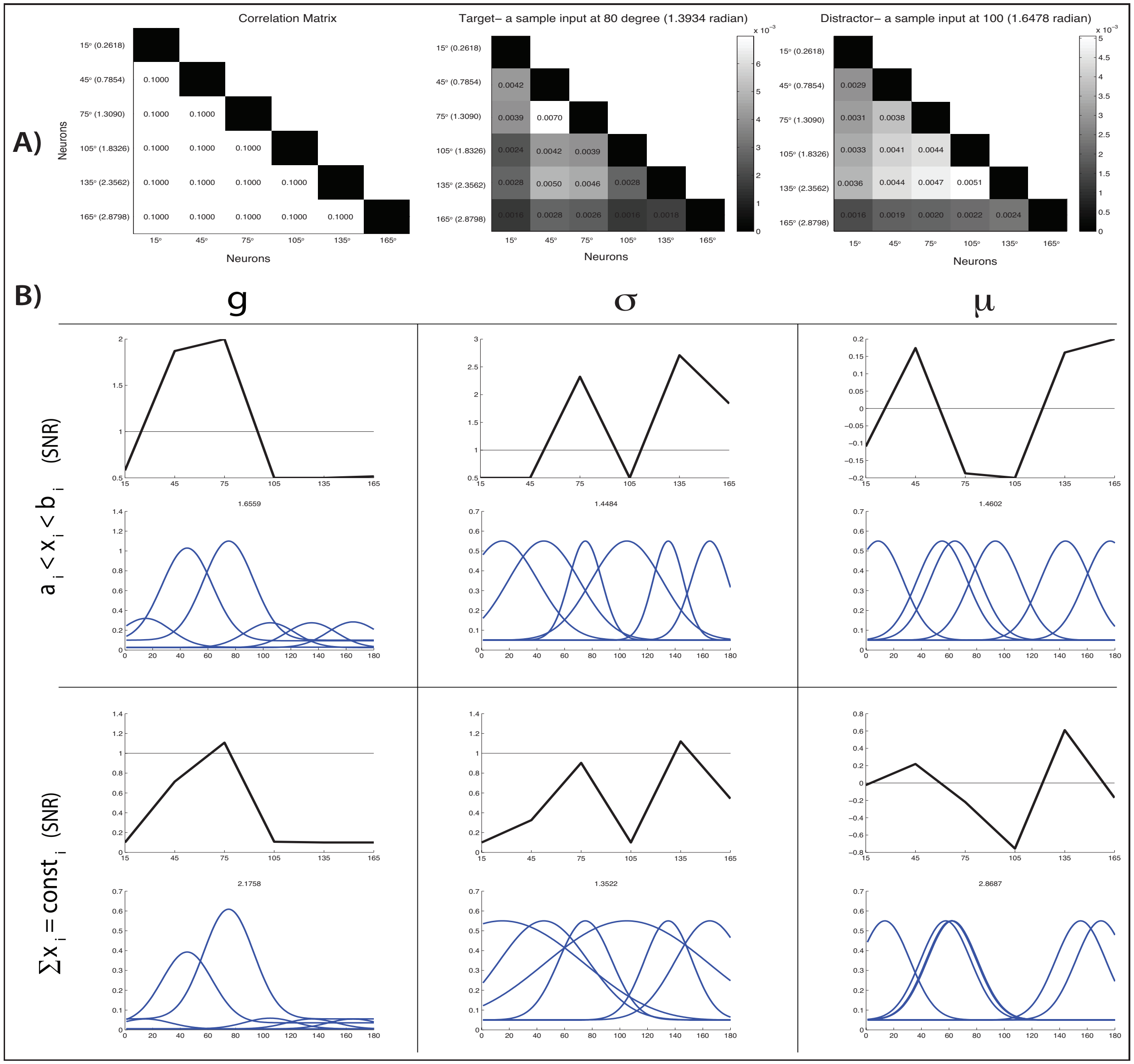

Figure 10: Simulation results over 6 neurons with correlated noise in visual search (target at 80, distractor at 100, $\sigma = 5$ corresponding to difficult task with low uncertainty in Fig. 2). A) Correlation coefficient matrix (for parameter c=0.1 in Berens et al. (2011)'s implementation), and population correlations for a sample draw from the target distribution at 1.3934 (radian) and a sample draw from the distractor distribution at 1.6478. Diagonal entries are set to 0 for better visualization. B) Optimal gain, tuning curve, and feature selectivity for constraint regimens 1 and 2. Compare these plots with their corresponding plots in Fig. 2.
